# The estrous cycle and 17β‐estradiol modulate the electrophysiological properties of rat nucleus accumbens core medium spiny neurons

**DOI:** 10.1111/jne.13122

**Published:** 2022-04-02

**Authors:** Amanda A. Krentzel, Stephanie B. Proaño, David M. Dorris, Beverly Setzer, John Meitzen

**Affiliations:** ^1^ Department of Biological Sciences North Carolina State University Raleigh NC USA; ^2^ Neurobiology Laboratory National Institute of Environmental Health Sciences, NIH Research Triangle Park NC USA; ^3^ Graduate Program for Neuroscience and Department of Biomedical Engineering Boston University Boston MA USA; ^4^ Comparative Medicine Institute North Carolina State University Raleigh NC USA; ^5^ Center for Human Health and the Environment North Carolina State University Raleigh NC USA

**Keywords:** estradiol, estrogen receptors, estrous cycle, nucleus accumbens, sex differences

## Abstract

The nucleus accumbens core is a key nexus within the mammalian brain for integrating the premotor and limbic systems and regulating important cognitive functions such as motivated behaviors. Nucleus accumbens core functions show sex differences and are sensitive to the presence of hormones such as 17β‐estradiol (estradiol) in normal and pathological contexts. The primary neuron type of the nucleus accumbens core, the medium spiny neuron (MSN), exhibits sex differences in both intrinsic excitability and glutamatergic excitatory synapse electrophysiological properties. Here, we provide a review of recent literature showing how estradiol modulates rat nucleus accumbens core MSN electrophysiology within the context of the estrous cycle. We review the changes in MSN electrophysiological properties across the estrous cycle and how these changes can be mimicked in response to exogenous estradiol exposure. We discuss in detail recent findings regarding how acute estradiol exposure rapidly modulates excitatory synapse properties in nucleus accumbens core but not caudate‐putamen MSNs, which mirror the natural changes seen across estrous cycle phases. These recent insights demonstrate the strong impact of sex‐specific estradiol action upon nucleus accumbens core neuron electrophysiology.

## 1 INTRODUCTION

A defining feature of endocrinology is the influence of gonadal hormones upon target tissues. The tissues targeted by gonadal hormones include the brain and its component cells in both males and females, with highly differential effects depending on developmental stage, sex, brain region, neuron type, and other variables, including local hormone synthesis.^1^ Gonadal hormones can both temporarily and permanently modulate target tissues from seconds to days to years. Here, we review the action of one gonadal hormone, 17β‐estradiol (estradiol), upon the electrophysiological properties of medium spiny neurons (MSNs). Estradiol can be a gonadal hormone, manufactured in the ovaries in females and the testes in males, and can also be produced in other organs such as the brain.^2^ Both of these sources are relevant to MSNs. Estradiol is also referenced as a steroid hormone, a sex hormone, and/or a sex steroid hormone.^3^ Estradiol acts across a wide timeframe,^4^ from years to days, to rapid and acute modulation in minutes to seconds.^5^, ^6^

Here, we focus upon estradiol action in the nucleus accumbens core in adult rats, in the context of biological sex and the estrous cycle. Estradiol triggers the activation of estrogen receptors (ERs). ERs can be located in various cellular compartments, including the nucleus and the plasma membrane. MSNs express nuclear ERα, ERβ, and GPER‐1 during early development. In adulthood, MSNs express plasma membrane‐associated ERα, ERβ, and GPER‐1.^7^, ^8^, ^9^, ^10^, ^11^, ^12^, ^13^, ^14^, ^15^, ^16^, ^17^, ^18^, ^19^ Thus, estradiol can alter MSN electrophysiological function and ultimately nucleus accumbens core output by binding to ERs on MSNs during both development and adulthood. In this review, we provide evidence supporting this theme. We first introduce the nucleus accumbens core and MSNs, and how MSN electrophysiological properties are differentiated by sex and sensitive to estradiol action. We then review the estrous cycle, and its impacts upon nucleus accumbens core MSN electrophysiological properties. We then discuss recent findings demonstrating how acute estradiol exposure rapidly and sex‐specifically modulates glutamatergic excitatory synapse properties in rat MSNs, and how these alterations mirror the natural changes seen across estrous cycle phases. We discuss how rapid estradiol action on glutamatergic synapses is not detected in rat caudate‐putamen. We conclude with a discussion of possible local estradiol synthesis, as well as future directions.

## 2 NUCLEUS ACCUMBENS CORE MSNS DIFFER BY SEX AND ARE SENSITIVE TO ESTRADIOL

The nucleus accumbens core is a striatal mesocorticolimbic region. In humans and rodents, the nucleus accumbens core serves as a critical nexus between limbic and premotor pathways to regulate motivated behaviors, especially the cognitive processing of reward and reinforcement.^20^, ^21^, ^22^, ^23^ The nucleus accumbens core and other striatal regions are evolutionarily ancient and well conserved across mammals, including rats.^24^, ^25^ In both humans and animal models, non‐pathological behaviors controlled by the nucleus accumbens core and other striatal regions exhibit sex differences and are modulated by hormones such as estradiol.^26^, ^27^, ^28^ Likewise, the phenotype and incidence of many disorders relevant to the nucleus accumbens core and the other striatal regions show sex differences and are modulated by hormones such as estradiol.^16^, ^29^, ^30^, ^31^, ^32^, ^33^, ^34^, ^35^, ^36^ These sex differences and estradiol sensitivity in non‐pathological behavior and relevant disorders make understanding the fundamental mechanisms underlying estradiol action in the nucleus accumbens core an important avenue of research. Of all the striatal regions, the nucleus accumbens core stands out as particularly sensitive to estradiol, as determined in diverse non‐electrophysiological contexts by multiple laboratory groups.^18^, ^33^, ^34^, ^37^, ^38^, ^39^, ^40^, ^41^, ^42^, ^43^, ^44^, ^45^, ^46^, ^47^, ^48^, ^49^, ^50^ The neurobiological mechanisms of how estradiol acts on neuron function in brain regions such as the nucleus accumbens core is in most cases underexplored. This situation is largely a result of the vast majority of preclinical research employing only males, with few studies directed at females, much less during different reproductive phases and estradiol levels.^51^, ^52^, ^53^, ^54^, ^55^ Therefore, it is important to understand whether and how estradiol can modulate nucleus accumbens core neuron electrophysiological properties. These findings not only benefit the understanding of fundamental neuroendocrinology, but also could possibly generate new personalized approaches for therapies of nucleus accumbens core‐related disorders informed by sex and hormone state.

**FIGURE 1:**
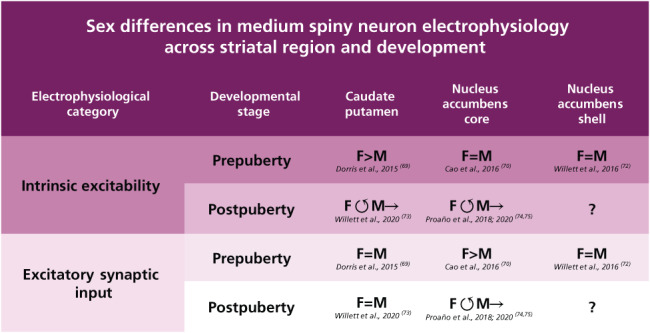
Developmental of rat medium spiny neuron electrophysiological properties in the context of striatal region, sex, and estrous cycle. ↺, indicates estrous cycle‐dependent sex differences are detected

The nucleus accumbens core's predominant neuron type is the GABAergic MSN. MSNs serve as the output neurons of the nucleus accumbens core, and integrate a wide array of inputs. These inputs include dopamine, which is the most famous and well‐studied input, especially in terms of estradiol action. Estradiol exposure typically augments dopaminergic action in a sex‐specific manner, as demonstrated by years of research from many laboratories.^18^, ^47^, ^56^, ^57^, ^58^, ^59^, ^60^ The role of estradiol and dopamine in the nucleus accumbens has been recently reviewed.^18^ The MSN also receives many other inputs, including inhibitory inputs from other MSNs and interneurons, and more prominently excitatory synaptic inputs from multiple brain regions that profoundly mediate function as excitatory synaptic inputs are essential for triggering MSN action potential production.^61^, ^62^, ^63^, ^64^, ^65^, ^66^, ^67^, ^68^ MSNs exhibit complex developmental period and region‐specific sex differences in both excitatory synapse electrophysiological properties^69^, ^70^, ^71^, ^72^, ^73^, ^74^, ^75^ (Figure 1), as well as in excitatory glutamatergic synapse‐related neuroanatomy. These neuroanatomical properties include synapse number, neurochemical markers associated with glutamatergic synapse, and dendritic spine density, most prominently in the nucleus accumbens core and halso in other striatal regions.^76^, ^77^, ^78^, ^79^, ^80^, ^81^ In the early 2010s, ultrastructure anatomical studies from Woolley and colleagues found increased glutamatergic synapse number in adult gonad‐intact female rat caudate‐putamen and nucleus accumbens when compared to males.^76^, ^78^ Supporting this finding, another study from the Woolley laboratory shows that gonad‐intact adult female rats exposed to cocaine demonstrated increased miniature excitatory postsynaptic current (mEPSC) frequencies compared to males treated with cocaine and female and male controls.^77^ mEPSC frequency, along with amplitude and decay, is commonly assessed excitatory synapse attributes.

These findings from Woolley and colleagues are foundational for establishing the sexually differentiated nature of glutamatergic neural circuitry in the nucleus accumbens. Soon after the publication of Wissman et al.,^77^ Cao et al.^70^ began testing whether prepubertal rat nucleus accumbens core MSNs showed sex differences in their electrophysiological properties, including glutamatergic synapse properties. They also tested whether perinatal exposure to masculinizing/defeminizing doses of estradiol and/or testosterone could alter glutamatergic excitatory neurotransmission in female rat nucleus accumbens, to mimic the natural masculinization process found in males. Cao et al.^70^ found, in prepubertal rats, that mEPSC frequency is higher in females compared to males. Changes in mEPSC frequency are typically associated with changes at the presynaptic side of the synapse, such as synapse number of differences in internal calcium sensitivity related to vesicle release probability. No sex differences in mEPSC amplitude or decay are detected prepuberty. mEPSC amplitude and decay is typically related to the properties of the postsynaptic side of the synapse, such as synaptic strength based upon AMPA receptor number and biophysical properties. The sex difference in mEPSC frequency is eliminated in females exposed to masculinizing/defeminizing doses of estradiol or testosterone as neonates.^70^ These findings indicate that sex differences in excitatory synaptic signaling onto MSNs in the nucleus accumbens core are present long before adulthood and are induced by hormone action during either the perinatal or prenatal periods. This organizational action by estradiol during early development then prepares the MSN to receive activational estradiol action upon mEPSC frequency and amplitude during the adult estrous cycle.

## 3 THE ESTROUS CYCLE

Reproductively active rodents, such as rats, and primates, such as humans, show cyclical fluctuations of hormones, including estradiol and progesterone, amongst others. In humans, cyclical fluctuations occur in ovarian production of estradiol and progesterone over the approximately 28‐day menstrual cycle, with peak circulating plasma levels of estradiol during the late follicular phase.^82^ Female rats exhibit an analogous approximately 4–5‐day estrous cycle, with similar but not identical fluctuations in ovarian hormones.^83^, ^84^, ^85^ Both cycles feature distinctive phases. Each phase, including metestrus, diestrus, proestrus, and estrus, exhibits distinct hormone concentration profiles, in addition to concomitant changes in reproductive organs, the brain, and associated behaviors.^83^, ^84^, ^85^, ^86^, ^87^, ^88^, ^89^ For example, the diestrus phase begins with relatively low levels of estradiol and progesterone that then gradually increase. The length of the diestrus phase can vary. Circulating levels of estradiol peak during the morning of proestrus phase (early proestrus, also called proestrus a.m.) and circulating levels of progesterone peak during the afternoon of proestrus phase (late proestrus, also called proestrus p.m.), which can exert differential actions on neuron function,^90^ including MSNs.^74^ Estrus phase is when select effects of estradiol and progesterone linger, even though the circulating concentrations of these hormones are low. Estradiol and progesterone action trigger distinct processes, and the combination of both is necessary for the expression of the full variety of mating behaviors. We also note that the estrous cycle is one natural cycle of many that modulates neural functions, and, although an important feature of neuroendocrinology and neurobiology, the ovarian cycle does not make females inherently more variable than males.^32^, ^91^, ^92^ Our focus in this review on the estrous cycle and 17β‐estradiol is meant to highlight one of many potent hormones that can have differential actions depending on the systemic states of sex and/or gender. Although most nucleus accumbens core hormone research has concentrated on estradiol,^17^, ^93^ including our own, there is evidence that circulating progesterone levels correlate with several nucleus accumbens core MSN excitatory synapse properties.^74^ In general, the estrous cycle and its associated hormones such as progesterone and estradiol are relevant for MSN and nucleus accumbens core function.

## 4 THE ESTROUS CYCLE INFLUENCES NUCLEUS ACCUMBENS CORE MSN ELECTROPHYSIOLOGY: ROLE OF ESTRADIOL

**FIGURE 2:**
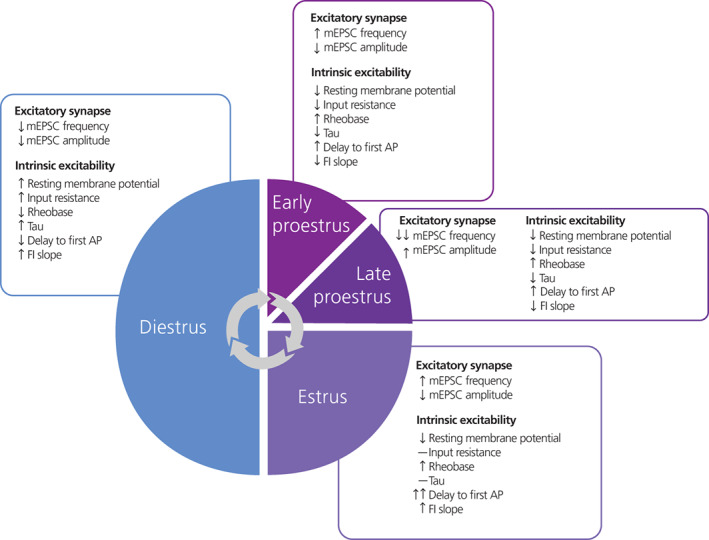
Schematic indicating changes in female rat nucleus accumbens core medium spiny neuron electrical properties across the estrous cycle. Changes in each attribute are depicted in comparison to an overall value over all cycle phases. Acronyms: ↑, increased value; ↓, decreased value; —, intermediate; AP, action potential; AP, action potential; FI, evoked firing rate‐to‐positive current curve; mEPSC, miniature excitatory postsynaptic current; Tau, time constant of the membrane; Input resistance, input resistance in the linear range. Input resistance in the rectified range follows a similar pattern. Not pictured on this schematic are the other following properties that change across the estrous cycle: inward rectification, input resistance in the rectified range, mEPSC decay, action potential width, and action potential afterhyperpolarization peak amplitude also differ across the estrous cycle. Data integrated from Proaño et al.^74^, ^75^

The estrous cycle has been shown to influence neuron physiology across multiple brain regions.^73^, ^75^, ^90^, ^94^, ^95^, ^96^, ^97^ Aside from the select regions already studied, we suspect that even more regions are sensitive to the estrous cycle than is now established as a result of the general lack of investigation into the neurobiology relevant to women's health. In naturally cycling female rats, MSNs show changes in both intrinsic excitability and excitatory synapse properties across estrous cycle phases, which generates sex differences compared to male MSNs^74^, ^75^ (Figure 2). Intrinsic excitability encompasses a variety of biophysical parameters that collectively embody the responsiveness of a neuron to electrical input. These parameters include such properties as resting membrane potential, and also properties such as action potential generation, rheobase, and input resistance, which are typically assessed by injecting neurons with electrical current and measuring changes in the output voltage.^98^ For example, a decreased input resistance (a measurement of the change in voltage to injected current), as well as an increased rheobase (the amount of current necessary to elicit an action potential), typically indicates decreased excitability. Changes in MSN excitability alter how a MSN responds to synaptic input and directly relate to changes in circuit output and behavior.^99^, ^100^, ^101^, ^102^, ^103^, ^104^, ^105^

MSNs recorded in early proestrus, late proestrus, and estrus phases showed hyperpolarized resting membrane potentials accompanied by decreases in input resistance and increases in rheobase compared to diestrus phase, among other select changes. These electrophysiological differences in intrinsic excitability properties are abolished when females are ovariectomized.^75^ They are restored upon exposure to an exogenous estradiol replacement paradigm,^106^ designed to mimic the changing temporal circulating levels of estradiol during the diestrus and proestrus phases of the rat estrous cycle.^107^ In our study, acute brain slices of nucleus accumbens core were made 2 h after the last injection of estradiol into the rat. Thus, MSNs were recorded during the equivalent of proestrus phase.^106^ In this preparation, MSNs exhibited decreased membrane excitability that was consistent with that demonstrated by MSNs in females in the early and late proestrus and estrus phases. MSNs did not exhibit membrane excitability consistent with that found in the diestrus phase. Consistent with MSNs recorded from the proestrus phase in naturally cycling females, MSNs recorded from the gonadectomized females receiving exogenous estradiol showed decreased input resistance in both the linear and rectified range, hyperpolarized resting membrane potential, and increased rheobase.^106^ These changes in intrinsic excitability induced by estradiol are hypothesized to be mediated by activation of membrane ERs expressed by MSNs, which in turn can act through intermediaries such as metabotropic glutamate receptors (MGluRs) to trigger intracellular signal cascades that ultimately modulate ion channel function and expression.^17^, ^108^ This longer, more genomic‐like action of membrane ERs could explain why the changes in intrinsic excitability persist for several hours after the last exposure to estradiol, as well as naturally during proestrus and estrus phases. What changes are induced in MSN ion channel expression and function by estradiol is not yet known, beyond L‐type calcium channel function.^12^ Given that estradiol induces a hyperpolarized resting membrane potential, accompanied by decreased input resistance, as well as increased rheobase, multiple biophysical mechanisms could underlie these changes. For example, estradiol could induce alterations in leak or inwardly rectifying (IRK) potassium channel expression, perhaps accompanied by changes in voltage‐gated and/or leak sodium channel expression. One working hypothesis is that IRK potassium channel and voltage gated sodium channel expression both change in response to estradiol exposure, but in opposite directions. Potassium channels have been shown to be modified by estradiol in other neuron types.^109^, ^110^, ^111^

Regarding excitatory synapse properties, mEPSC frequency, amplitude, and decay robustly differed across the estrous cycle. Specifically, mEPSC frequency increased during early proestrus and estrus phases compared to diestrus phase, and significantly decreased in late proestrus phase compared to all other phases.^74^, ^75^ This drop in mEPSC frequency in late proestrus phase is unrivaled in magnitude, and we consider this represents the largest natural decrease in mEPSC frequency ever reported in the literature. Interestingly, although mEPSC frequency falls, MSNs in late proestrus phase maintain intrinsic excitability comparable to that measured in early proestrus and estrus. This curtailment of excitatory synapse activity is mediated by a rapid onset and acute activational effect of estradiol,^112^ and also by a potential synergistic action with progesterone.^74^ A common anatomical correlate of changes in mEPSC frequency are changes in dendritic spine density. It is not yet known whether MSN hdendritic spine properties naturally change across the estrous cycle, although pilot data from our laboratory supports this conclusion. In adult gonadectomized female rats and hamsters, a series of estradiol injections decreases nucleus accumbens core dendritic spine density by activating metabotropic glutamate receptor 5 (mGluR5) and endocannabinoid signaling.^11^, ^12^, ^80^, ^81^, ^113^ No sex differences or estradiol effects have been detected on other anatomical attributes such as MSN soma size, density or striatal region volume.^114^, ^115^

By contrast to mEPSC frequency, mEPSC amplitude is significantly increased in late proestrus phase compared to the diestrus, early proestrus, and estrus phases of the cycle. Regarding mEPSC decay, there are also significant differences across the estrous cycle that are quantitatively established to be the product of changes in mEPSC amplitude. These electrophysiological differences in excitatory synapse properties are abolished when females are ovariectomized,^75^ and are consistent with a previous study comparing proestrus phase female and male nucleus accumbens core MSNs.^77^ These changes in mEPSC properties are not restored using the exogenous estradiol replacement paradigm.^106^ There are several possible explanations for this lack of restoration, with the most salient being that estradiol action on nucleus accumbens core MSN excitatory synapse properties is regulated by the rapid action of estradiol that acutely modulates and maintains the suppression of excitatory synapse properties such as mEPSC frequency.^112^ Such a rapid action of estradiol may not be detected by the technique employed in the exogenous estradiol replacement study,^106^ given that animals are sacrificed 2 h after the last estradiol injection. A temporal difference in the impact of estradiol on electrophysiological properties, especially between the seconds to minutes to hours timescale, has been demonstrated in other systems.^109^ There may also be differences in MSN electrophysiology in response to repeated doses of estradiol because several nucleus accumbens core related behaviors are sensitive to estrogen priming.^116^, ^117^, ^118^ It is unknown whether inhibitory synaptic inputs and synaptic plasticity change across the estrous cycle.

There are at least two competing interpretations for the changes in MSN excitatory synapse and intrinsic excitability properties across the estrous cycle. First, estrous cycle sensitive properties directly facilitate related changes in nucleus accumbens core function resulting in altered behaviors. A second interpretation posits that changes in excitatory synaptic input and intrinsic excitability compensate for each other to mitigate differential circuit output, perhaps via an estrous cycle associated homeostatic plasticity mechanism, as detailed in other circumstances.^119^, ^120^ This second interpretation is based upon potentially compensatory changes in excitatory synapse properties and intrinsic excitability between diestrus phase MSNs and early proestrus and estrus phases MSNs. Arguing against this possibility, the late proestrus phase of the estrous cycle demonstrates different patterns of nucleus accumbens core MSN electrophysiological properties compared to other phases of the cycle, corresponding with this phase's differential hormonal, behavioral, and reproductive functions. The potentially compensatory changes in excitatory synapse properties and intrinsic excitability exhibited between diestrus phase compared to early proestrus and estrus phases dissipated during late proestrus phase. During late proestrus phase, changes in excitatory synapse properties are disengaged from changes in intrinsic excitability, and vice versa. Another line of evidence arguing against homeostatic plasticity is that different estradiol exposures result in differential effects on MSN electrophysiological properties, as well as the differential relationship of MSN electrophysiological properties to circulating levels of either estradiol, progesterone, or the combination of both, as may occur during the estrus phase.^74^, ^106^, ^112^ There are also varying timeframes by which electrophysiological properties remain altered in response to estrous cycle phase. For example, several electrophysiological properties during the estrus phase display a more diestrus phase‐like phenotype, others assume a more proestrus phase‐like (both early and late) phenotype, while others exhibit more intermediate values (Figure 2). Overall, the specific alterations in MSN electrophysiological properties across the estrous cycle are dissociable and inducible by differential estradiol (and likely progesterone) exposures. We favor the first interpretation: changing electrophysiological properties across the estrous cycle directly facilitate changes in nucleus accumbens core function, mostly regarding its role in motivated reproductive behaviors.^121^ These behaviors could potentially include the changes in sexual receptivity detected during the estrous cycle,^18^, ^89^ paced mating behavior,^122^ sexual reward,^123^ sensorimotor locomotor, and exploratory/anxiety‐related behaviors,^36^, ^117^, ^118^, ^124^ and, at least in some species, mate choice and pair bonding.^125^, ^126^ We do note that correlation is not causation, and an important future direction will be to establish causal roles of estrous cycle action and ovarian hormone effects in the nucleus accumbens core to specific behavioral phenotypes.

## 5 ESTRADIOL RAPIDLY MODULATES EXCITATORY SYNAPSE PROPERTIES IN A SEX‐SPECIFIC MANNER IN RAT NUCLEUS ACCUMBENS CORE BUT NOT CAUDATE‐PUTAMEN

**FIGURE 3:**
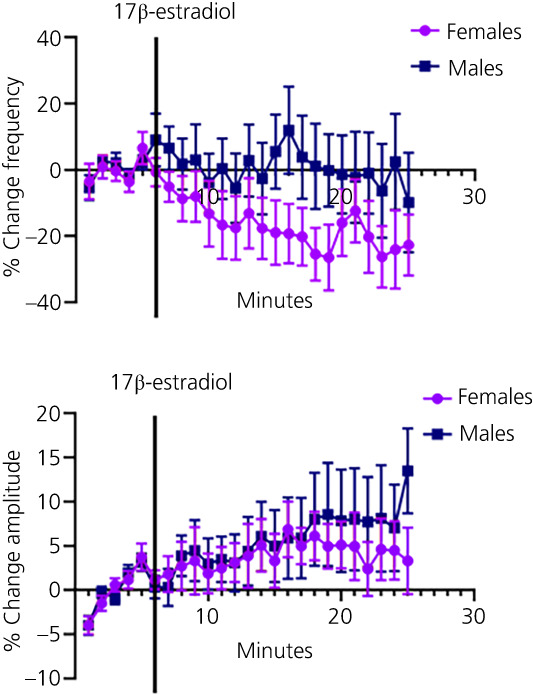
Estradiol rapidly modulates excitatory synapse properties in nucleus accumbens core medium spiny neurons within minutes of exposure. Top: Plotting the normalized change in miniature excitatory postsynaptic current (mEPSC) frequency versus time indicates that adult female rat nucleus accumbens core medium spiny neurons (MSNs) (*n* = 16) are robustly sensitive to rapid estradiol action upon mEPSC frequency while adult male nucleus accumbens core MSNs are not (*n* = 14). Bottom: Plotting the normalized change in mEPSC amplitude versus time indicates that adult female (*n* = 16) and male (*n* = 14) rat nucleus accumbens core MSNs are weakly sensitive to rapid estradiol action upon mEPSC amplitude. Vertical line indicates application of 100 nm 17β‐estradiol. Vehicle alone controls showed no effect on either mEPSC frequency or amplitude (data not shown). Estradiol application exerts no effect on mEPSC frequency or mEPSC amplitude in either female or male MSNs recorded from the adult rat caudate‐putamen. Data replotted from Krentzel et al.^112^

Consistent with the hypothesis that estradiol acutely regulates a suppression of excitatory synapse properties, estradiol rapidly modulates nucleus accumbens core MSN excitatory synapse properties within minutes of exposure in adult female rats^112^ (Figure 3). This action of estradiol is present in MSNs recorded in the nucleus accumbens core but not the caudate‐putamen, and shows sex‐specificity in its actions upon mEPSC frequency but not mEPSC amplitude. mEPSC frequency robustly decreases in response to estradiol in female MSNs recorded across estrous phases but not male MSNs. To our knowledge, this is the first demonstration of an acute action of estradiol on excitatory synapse properties upon MSNs in any striatal region. The decrease in mEPSC frequency in response to estradiol does not correlate with any MSN intrinsic electrical property. Considering that mEPSC frequency is primarily associated with changes in presynaptic properties (i.e., number of synapses and/or neurotransmitter release), it is logical that MSN intrinsic properties do not relate to mEPSC frequency.

mEPSC amplitude moderately increases in response to estradiol in both male and female MSNs^112^ (Figure 3). This change in mEPSC amplitude is considerably smaller in magnitude than that observed in mEPSC frequency. Thus, overall, acute estradiol exposure engenders a decrease in glutamatergic excitatory neurotransmission onto female MSNs, whereas, in males, if anything, it subtly augments excitatory neurotransmission. The protocol used in our laboratory to record mEPSCs specifically targets those generated by activation of glutamatergic AMPA channels.^75^ The increase in mEPSC amplitude generated by acute estradiol exposure is a result of amplified AMPA receptor signaling. This augmentation could occur via AMPA receptor trafficking to the membrane, phosphorylation or some other mechanism. MSN dendritic‐expressed estrogen receptor α may directly facilitate this effect. Pilot experiments from our laboratory support this conclusion, although these data remains to be published. MSNs exhibiting concurrent lower rheobase values and more hyperpolarized action potential thresholds show the greatest positive percent change in mEPSC amplitude in response to estradiol. Collectively, these data indicate that increased MSN excitability, as measured by a reduced rheobase and action potential threshold, associates with increased likelihood of estradiol‐sensitivity in mEPSC amplitude. Rheobase is a strong predictor of whether a MSN is a D1 or D2 MSN subtype (D1 > D2; as reviewed previously^127^, ^128^), however, these strict definitions of MSN subtypes tend to break down in the nucleus accumbens core.^129^ Sex differences and estradiol sensitivity between MSN subtypes remains an active avenue of research.^127^, ^130^ These data predict that D2 subtype MSNs exhibit enhanced sensitivity to estradiol action upon mEPSC amplitude. Further testing with D1 or D2 labeled neurons in a relevant animal model must be conducted to confirm this prediction. Because rheobase or any other property fails to correlate with the impact of estradiol upon mEPSC frequency, it is likely that both MSN subtypes are sensitive to estradiol action upon mEPSC frequency.

The detected decrease in mEPSC frequency and amplitude likely indicates a locus of action that modulates the presynaptic terminal and postsynaptic dendrite respectively. Regarding changes in mEPSC frequency, it is possible that estradiol could be acting either directly on the presynaptic glutamatergic synaptic terminals or acting on the MSN (or perhaps even some other neuron type local to that MSN in the nucleus accumbens core) to trigger a retrograde messenger. Consistent with retrograde signaling, endocannabinoid action mediates estradiol modulation of MSN dendritic spine density in the nucleus accumbens core and associates with membrane ER and mGluR action.^113^ GPER‐1 is also expressed in the nucleus accumbens core and other striatal regions and could also potentially play a role.^14^, ^19^, ^131^ There is evidence from the hippocampus that sex‐specific ERs can play key roles in regulating both presynaptic and postsynaptic glutamatergic transmission,^132^ and this may be occurring in the nucleus accumbens core, given that both mEPSC frequency and amplitude are modulated by estradiol in distinct sex‐specific manners. ERα, ERβ and GPER1 expression are present in the nucleus accumbens core on terminals and dendrites of MSNs of female rats.^7^, ^10^, ^14^, ^19^ Sex differences in ER expression on glutamatergic terminals may mediate the sex differences in the estradiol sensitivity to presynaptic excitatory synapse properties. To date, these sex‐specific comparisons have not been conducted.

## 6 ESTRADIOL DID NOT RAPIDLY MODULATE EXCITATORY SYNAPSE PROPERTIES IN CAUDATE‐PUTAMEN MSNS

It is highly significant that estradiol application does not rapidly modulate mEPSC properties in either female or male caudate‐putamen MSNs.^112^ This negative finding does not mean that caudate‐putamen MSNs are insensitive to acute estradiol exposure. Indeed, at this point, it is still the case that the majority of studies of striatal estradiol action have been performed in the caudate‐putamen, although this situation is changing as the sensitivity of the nucleus accumbens core to estradiol becomes more prominent.^17^ There is one broad finding from this body of research in the caudate‐putamen that is consistent with our work on rapid estradiol modulation of nucleus accumbens core MSN: female MSNs appear to be more sensitive to acute estradiol action than are male MSNs.^12^, ^18^, ^133^ Two different studies dating from the 1980s demonstrate differences in the in vivo spontaneous action potential generation rates of MSNs across different phases of the estrous cycle and in response to longer‐term exposure to exogenous estradiol exposure in ovariectomized female rat caudate‐putamen.^134^, ^135^ Arnauld et al.^135^ made the initial discovery that estradiol exposure augmented dopamine sensitivity and in vivo spontaneous action potential firing rates in adult female ovariectomized rat caudate‐putamen. However, this finding can not be classified as rapid estradiol action per se given the timing of the exogenous estradiol exposure. Tansey et al.^134^ followed up on this study to show that the in vivo spontaneous action potential firing rates of identified striatonigral MSNs are augmented in the caudate‐putamen of adult female rats during the high‐estrogen phases of the estrous cycle or in response to estradiol implants compared to the low‐estrogen phases of the estrous cycle or males. The study by Tansey et al.^134^ is the first to demonstrate a sex‐specific estradiol action upon striatal MSNs and is also the first to show that the electrophysiological properties of MSNs could change within hours as part of the estrous cycle.

The field of estradiol action upon striatal neuron electrophysiology then lay dormant for over a decade, until the mid‐1990s. In a groundbreaking study, Mermelstein et al.^12^ showed that, in female but not male rat caudate‐putamen MSNs, estradiol decreased L‐type calcium channel currents in a membrane‐associated ER. The effect of estradiol on the L‐type calcium channel is likely not directly responsible for the changes in caudate‐putamen MSN excitability noted in the studies conducted in the 1980s^134^, ^135^ or more contemporary research from our own laboratory.^69^, ^73^, ^136^ This conclusion is a result of the L‐type calcium channel's primary role in triggering internal signaling cascades typically activated by sustained depolarization.^137^ Alterations in L‐type calcium channel conductance did not robustly alter intrinsic excitability in a computational study of MSN biophysics conducted in our laboratory. Grove‐Strawser et al.^11^ later linked this decrease in L‐type calcium channel currents to modulation of a signal transduction cascade that modulated CREB phosphorylation in MSNs, along with identifying the specific activated estrogen receptor as membrane ERβ. This membrane ERβ activates a mGluR, which is important given that membrane ERβ itself is not a GPCR. Other studies demonstrate that acute exposure to estradiol can induce not only behavioral changes, but also decreases in GABA release and increases in dopamine transmission in the caudate‐putamen after an acute estradiol injection.^56^, ^57^, ^138^

## 7 ENDOGENOUS ESTRADIOL PRODUCTION IN THE NUCLEUS ACCUMBENS

Although this review focuses upon gonadal estradiol acting in the context of the estrous cycle, the production of endogenous estradiol within the nucleus accumbens core may also play a prominent role. A previous review provides sufficient evidence to conclude that the striatal regions are potentially capable of producing local estradiol,^36^ consistent with supporting data of aromatase protein expression across striatal brain regions including nucleus accumbens core.^19^ Functionally, Tozzi et al.^139^ detected that aromatase activity is necessary for at least one type of long‐term potentiation (LTP) in adult male caudate‐putamen MSNs. Female and nucleus accumbens core MSNs were not tested. Tozzi et al.^139^ found that additional estradiol application did not augment LTP, suggesting that there may already be enough endogenously produced estradiol in the striatal regions to modulate neural physiology. Aromatase inhibition experiments will be an important avenue of future experiments for the nucleus accumbens core in both males and females, reminiscent of previous experiments in the songbird model organism. Work in the zebra finch, an oscine songbird, indicated that brain regions featuring enriched aromatase exhibit sensitivity to rapid estradiol modulation of neural electrophysiology.^140^ Remage‐Healey^140^ have also demonstrated that neurons in brain regions that receive projections from areas with enriched aromatase can also be rapidly modulated. This indirect mechanism could be occurring in the nucleus accumbens core, as trans‐synaptic hormone action occurs from cortical‐like brain regions to striatal regions in white‐crowned sparrow songbird brain.^141^, ^142^ However, the cell bodies of the neurons providing the glutamatergic synaptic terminals are not present in the employed nucleus accumbens core brain slice preparation, suggesting that estradiol action is specific to either the presynaptic terminal or triggers retrograde signals originating from the MSN (or potentially some other neuron). Related to this point, multiple types of membrane ERs are present on both the pre‐ and postsynaptic sides of glutamatergic synapse onto striatal MSNs in general, and also in striatal cholinergic and dopaminergic synapses.^7^, ^10^, ^14^ There is evidence that estradiol can modulate striatal cholinergic systems.^10^, ^143^ In any case, inhibiting aromatase action and ER blockade both comprise critical future experiments with the aim of determining the underlying mechanism of rapid estradiol action upon nucleus accumbens core MSNs.

## 8 FUTURE DIRECTIONS

The mechanisms underlying all of these estradiol actions and their interrelations between themselves and the action of other hormones are all clear and important avenues for future investigations. The identity of the underlying sex‐specific ERs is a key question, as are the consequential signaling cascades. Males and females may utilize different ER types and these ERs may differ in mediating either presynaptic or postsynaptic changes, as has been shown in the adult hippocampus^132^ and prepubertal caudate‐putamen.^136^ How ER expression changes in response to the adult female hormone cycle and to relevant environmental and pathological contexts remains unexplored.^19^ Whether ER expression and consequent action differs between MSN subtype remains an open question, as is estradiol action upon striatal neuron subtypes such as the cholinergic interneurons. A differential distribution of ERs by either sex, endocrine status or subtype would exert profound effects regarding nucleus accumbens function and relevant disorders.

## 9 CONCLUSIONS

It is clear that the estrous cycle modulates nucleus accumbens core MSNs and that estradiol is an important component of this action. As befits a complex, natural process, select aspects of changes induced by the estrous cycle regarding MSN intrinsic excitability are mimicked by estradiol action over a slower, more persistent time course, and some aspects regarding MSN excitatory synapse properties are mimicked by estradiol action over a rapid time course. Given the strong influence of sex and hormones upon nucleus accumbens core‐relevant behaviors and disorders,^34^, ^144^, ^145^ the role of estradiol in modulating nucleus accumbens core neuron function in all its respects represents an important avenue of future research.

## AUTHOR CONTRIBUTIONS

**Amanda Krentzel:** Visualization; writing – original draft; writing – review and editing. **Stephanie Proano:** Visualization; writing – original draft; writing – review and editing. **David Dorris:** Visualization; writing – review and editing. **Beverly Setzer:** Formal analysis; investigation; methodology; writing – review and editing. **John Meitzen:** Conceptualization; funding acquisition; project administration; supervision; visualization; writing – original draft; writing – review and editing.

## CONFLICTS OF INTEREST

The authors declare that they have no conflicts of interest.

### PEER REVIEW

The peer review history for this article is available at https://publons.com/publon/10.1111/jne.13122.

## Data Availability

Data sharing is not applicable to this article as no new data were created or analyzed in this study.

## References

[1] Schmidt KL , Pradhan DS , Shah AH , Charlier TD , Chin EH , Soma KK . Neurosteroids, immunosteroids, and the balkanization of endocrinology. Gen Comp Endocrinol. 2008;157(3):266‐274. 18486132 10.1016/j.ygcen.2008.03.025

[2] Remage‐Healey L . Frank Beach award winner: steroids as neuromodulators of brain circuits and behavior. Horm Behav. 2014;66(3):552‐560. 25110187 10.1016/j.yhbeh.2014.07.014 PMC4180446

[3] Wierman ME . Sex steroid effects at target tissues: mechanisms of action. Adv Physiol Educ. 2007;31(1):26‐33. 17327579 10.1152/advan.00086.2006

[4] Rudolph LM , Cornil CA , Mittelman‐Smith MA , et al. Actions of steroids: new neurotransmitters. J Neurosci. 2016;36(45):11449‐11,458. 27911748 10.1523/JNEUROSCI.2473-16.2016 PMC5125212

[5] Kelly MJ , Moss RL , Dudley CA . Differential sensitivity of preoptic‐septal neurons to microelectrophoresed estrogen during the estrous cycle. Brain Res. 1976;114(1):152‐157. 986858 10.1016/0006-8993(76)91017-9

[6] Szego CM , Davis JS . Adenosine 3′,5′‐monophosphate in rat uterus: acute elevation by estrogen. Proc Natl Acad Sci U S A. 1967;58(4):1711‐1718. 4295833 10.1073/pnas.58.4.1711 PMC223984

[7] Almey A , Milner TA , Brake WG . Estrogen receptors in the central nervous system and their implication for dopamine‐dependent cognition in females. Horm Behav. 2015;74:125‐138. 26122294 10.1016/j.yhbeh.2015.06.010 PMC4820286

[8] Foidart AN , Harada N , Balthazart J . Aromatase‐immunoreactive cells are present in mouse brain areas that are known to express high levels of aromatase activity. Cell Tissue Res. 1995;280(3):561‐574. 7606769 10.1007/BF00318360

[9] Stanic D , Dubois S , Chua HK , et al. Characterization of aromatase expression in the adult male and female mouse brain. I. Coexistence with oestrogen receptors alpha and beta, and androgen receptors. PLoS One. 2014;9(3):e90451. 24646567 10.1371/journal.pone.0090451 PMC3960106

[10] Almey A , Filardo EJ , Milner TA , Brake WG . Estrogen receptors are found in glia and at Extranuclear neuronal sites in the dorsal striatum of female rats: evidence for cholinergic but not dopaminergic colocalization. Endocrinology. 2012;153:5373‐5383. 22919059 10.1210/en.2012-1458 PMC3473205

[11] Grove‐Strawser D , Boulware MI , Mermelstein PG . Membrane estrogen receptors activate the metabotropic glutamate receptors mGluR5 and mGluR3 to bidirectionally regulate CREB phosphorylation in female rat striatal neurons. Neuroscience. 2010;170:1045‐1055. 20709161 10.1016/j.neuroscience.2010.08.012 PMC2949475

[12] Mermelstein PG , Becker JB , Surmeier DJ . Estradiol reduces calcium currents in rat neostriatal neurons via a membrane receptor. J Neurosci. 1996;16(2):595‐604. 8551343 10.1523/JNEUROSCI.16-02-00595.1996 PMC6578633

[13] Schultz KN , von Esenwein SA , Hu M , et al. Viral vector‐mediated overexpression of estrogen receptor‐alpha in striatum enhances the estradiol‐induced motor activity in female rats and estradiol‐modulated GABA release. J Neurosci. 2009;29(6):1897‐1903. 19211896 10.1523/JNEUROSCI.4647-08.2009 PMC2655639

[14] Almey A , Milner TA , Brake WG . Estrogen receptor alpha and G‐protein coupled estrogen receptor 1 are localized to GABAergic neurons in the dorsal striatum. Neurosci Lett. 2016;622:118‐123. 27080432 10.1016/j.neulet.2016.04.023 PMC5104174

[15] Le Saux M , Morissette M , Di Paolo T . ERbeta mediates the estradiol increase of D2 receptors in rat striatum and nucleus accumbens. Neuropharmacology. 2006;50(4):451‐457. 16309717 10.1016/j.neuropharm.2005.10.004

[16] Becker JB , Hu M . Sex differences in drug abuse. Front Neuroendocrinol. 2008;29(1):36‐47. 17904621 10.1016/j.yfrne.2007.07.003 PMC2235192

[17] Meitzen J , Meisel RL , Mermelstein PG . Sex differences and the effects of estradiol on striatal function. Curr Opin Behav Sci. 2018;23:42‐48. 30221186 10.1016/j.cobeha.2018.03.007 PMC6136839

[18] Yoest KE , Quigley JA , Becker JB . Rapid effects of ovarian hormones in dorsal striatum and nucleus accumbens. Horm Behav. 2018;104:119‐129. 29626485 10.1016/j.yhbeh.2018.04.002 PMC6197937

[19] Krentzel AA , Willett JA , Johnson AG , Meitzen J . Estrogen receptor alpha, G‐protein coupled estrogen receptor 1, and aromatase: developmental, sex, and region‐specific differences across the rat caudate‐putamen, nucleus accumbens core and shell. J Comp Neurol. 2021;529(4):786‐801. 32632943 10.1002/cne.24978 PMC7775873

[20] Floresco SB . The nucleus accumbens: an interface between cognition, emotion, and action. Annu Rev Psychol. 2015;66:25‐52. 25251489 10.1146/annurev-psych-010213-115159

[21] Francis TC , Lobo MK . Emerging role for nucleus Accumbens medium spiny neuron subtypes in depression. Biol Psychiatry. 2017;81(8):645‐653. 27871668 10.1016/j.biopsych.2016.09.007 PMC5352537

[22] Salgado S , Kaplitt MG . The nucleus Accumbens: a comprehensive review. Stereotact Funct Neurosurg. 2015;93(2):75‐93. 25720819 10.1159/000368279

[23] Yager LM , Garcia AF , Wunsch AM , Ferguson SM . The ins and outs of the striatum: role in drug addiction. Neuroscience. 2015;301:529‐541. 26116518 10.1016/j.neuroscience.2015.06.033 PMC4523218

[24] Do J , Kim JI , Bakes J , Lee K , Kaang BK . Functional roles of neurotransmitters and neuromodulators in the dorsal striatum. Learn Mem. 2012;20(1):21‐28. 23247251 10.1101/lm.025015.111

[25] Rolls ET . Neurophysiology and cognitive functions of the striatum. Rev Neurol (Paris). 1994;150(8–9):648‐660. 7754303

[26] Hampson E . Estrogen‐related variations in human spatial and articulatory‐motor skills. Psychoneuroendocrinology. 1990;15(2):97‐111. 2359813 10.1016/0306-4530(90)90018-5

[27] Kent S , Hurd M , Satinoff E . Interactions between body temperature and wheel running over the estrous cycle in rats. Physiol Behav. 1991;49(6):1079‐1084. 1896490 10.1016/0031-9384(91)90334-k

[28] Marcondes FK , Miguel KJ , Melo LL , Spadari‐Bratfisch RC . Estrous cycle influences the response of female rats in the elevated plus‐maze test. Physiol Behav. 2001;74(4–5):435‐440. 11790402 10.1016/s0031-9384(01)00593-5

[29] Wickens MM , Bangasser DA , Briand LA . Sex differences in psychiatric disease: a focus on the glutamate system. Front Mol Neurosci. 2018;11:197. 29922129 10.3389/fnmol.2018.00197 PMC5996114

[30] Picillo M , Nicoletti A , Fetoni V , Garavaglia B , Barone P , Pellecchia MT . The relevance of gender in Parkinson's disease: a review. J Neurol. 2017;264(8):1583‐1607. 28054129 10.1007/s00415-016-8384-9

[31] Young KA , Gobrogge KL , Liu Y , Wang Z . The neurobiology of pair bonding: insights from a socially monogamous rodent. Front Neuroendocrinol. 2011;32(1):53‐69. 20688099 10.1016/j.yfrne.2010.07.006 PMC3012750

[32] Becker JB , Prendergast BJ , Liang JW . Female rats are not more variable than male rats: a meta‐analysis of neuroscience studies. Biol Sex Differ. 2016;7:34. 27468347 10.1186/s13293-016-0087-5 PMC4962440

[33] Carroll ME , Anker JJ . Sex differences and ovarian hormones in animal models of drug dependence. Horm Behav. 2010;58(1):44‐56. 19818789 10.1016/j.yhbeh.2009.10.001

[34] Lorsch ZS , Loh YE , Purushothaman I , et al. Estrogen receptor alpha drives pro‐resilient transcription in mouse models of depression. Nat Commun. 2018;9(1):1116. 29549264 10.1038/s41467-018-03567-4 PMC5856766

[35] Becker JB , Chartoff E . Sex differences in neural mechanisms mediating reward and addiction. Neuropsychopharmacology. 2019;44(1):166‐183. 29946108 10.1038/s41386-018-0125-6 PMC6235836

[36] Krentzel AA , Meitzen J . Biological sex, estradiol and striatal medium spiny neuron physiology: a mini‐review. Front Cell Neurosci. 2018;12:492. 30618639 10.3389/fncel.2018.00492 PMC6299026

[37] Beatty WW . Gonadal hormones and sex differences in nonreproductive behaviors in rodents: organizational and activational influences. Horm Behav. 1979;12(2):112‐163. 573741 10.1016/0018-506x(79)90017-5

[38] Becker, J.B. Hormonal influences on sensorimotor function. In: J.B. Becker , Breedlove, S.M. , Crews, D. , McCarthy, M. M. , eds. Behavioral Endocrinology. 2002, MIT Press. p. 497–525.

[39] Bobzean SA , DeNobrega AK , Perrotti LI . Sex differences in the neurobiology of drug addiction. Exp Neurol. 2014;259C:64‐74. 10.1016/j.expneurol.2014.01.022 24508560

[40] Davis DM , Jacobson TK , Aliakbari S , Mizumori SJ . Differential effects of estrogen on hippocampal‐ and striatal‐dependent learning. Neurobiol Learn Mem. 2005;84(2):132‐137. 16054404 10.1016/j.nlm.2005.06.004

[41] Fattore L , Melis M , Fadda P , Fratta W . Sex differences in addictive disorders. Front Neuroendocrinol. 2014;35:272‐284. 24769267 10.1016/j.yfrne.2014.04.003

[42] Pavon JM , Whitson HE , Okun MS . Parkinson's disease in women: a call for improved clinical studies and for comparative effectiveness research. Maturitas. 2010;65(4):352‐358. 20117891 10.1016/j.maturitas.2010.01.001 PMC2875870

[43] Zurkovsky L , Brown SL , Boyd SE , Fell JA , Korol DL . Estrogen modulates learning in female rats by acting directly at distinct memory systems. Neuroscience. 2007;144(1):26‐37. 17052857 10.1016/j.neuroscience.2006.09.002 PMC1931581

[44] Di Paolo T . Modulation of brain dopamine transmission by sex steroids. Rev Neurosci. 1994;5(1):27‐41. 8019704 10.1515/revneuro.1994.5.1.27

[45] Becker JB , Perry AN , Westenbroek C . Sex differences in the neural mechanisms mediating addiction: a new synthesis and hypothesis. Biol Sex Differ. 2013;3(1):14. 10.1186/2042-6410-3-14 PMC3724495 22676718

[46] Walker QD , Ray R , Kuhn CM . Sex differences in neurochemical effects of dopaminergic drugs in rat striatum. Neuropsychopharmacology. 2006;31(6):1193‐1202. 16237396 10.1038/sj.npp.1300915

[47] Calipari ES , Juarez B , Morel C , et al. Dopaminergic dynamics underlying sex‐specific cocaine reward. Nat Commun. 2017;8:13877. 28072417 10.1038/ncomms13877 PMC5234081

[48] Bonansco C , Martínez‐Pinto J , Silva RA , et al. Neonatal exposure to oestradiol increases dopaminergic transmission in nucleus accumbens and morphine‐induced conditioned place preference in adult female rats. J Neuroendocrinol. 2018;30(7):e12574. 29377365 10.1111/jne.12574

[49] Alonso‐Caraballo Y , Ferrario CR . Effects of the estrous cycle and ovarian hormones on cue‐triggered motivation and intrinsic excitability of medium spiny neurons in the nucleus Accumbens core of female rats. Horm Behav. 2019;116:104583. 31454509 10.1016/j.yhbeh.2019.104583 PMC7256930

[50] Kopec AM , Smith CJ , Ayre NR , Sweat SC , Bilbo SD . Microglial dopamine receptor elimination defines sex‐specific nucleus accumbens development and social behavior in adolescent rats. Nat Commun. 2018;9(1):3769. 30254300 10.1038/s41467-018-06118-z PMC6156594

[51] Beery AK , Zucker I . Sex bias in neuroscience and biomedical research. Neurosci Biobehav Rev. 2011;35(3):565‐572. 20620164 10.1016/j.neubiorev.2010.07.002 PMC3008499

[52] Tannenbaum C , Ellis RP , Eyssel F , Zou J , Schiebinger L . Sex and gender analysis improves science and engineering. Nature. 2019;575(7781):137‐146. 31695204 10.1038/s41586-019-1657-6

[53] Galea LAM , Choleris E , Albert AYK , McCarthy MM , Sohrabji F . The promises and pitfalls of sex difference research. Front Neuroendocrinol. 2020;56:100817. 31837339 10.1016/j.yfrne.2019.100817 PMC7050281

[54] Mamlouk GM , Dorris DM , Barrett LR , Meitzen J . Sex bias and omission in neuroscience research is influenced by research model and journal, but not reported NIH funding. Front Neuroendocrinol. 2020;57:100835. 32070715 10.1016/j.yfrne.2020.100835 PMC7225067

[55] Will TR , Proaño SB , Thomas AM , et al. Problems and progress regarding sex bias and omission in neuroscience research. eNeuro. 2017;4(6):ENEURO.0278‐17.2017. 10.1523/ENEURO.0278-17.2017 PMC5677705 29134192

[56] Becker JB . Direct effect of 17 beta‐estradiol on striatum: sex differences in dopamine release. Synapse. 1990;5(2):157‐164. 2309159 10.1002/syn.890050211

[57] Becker JB . Gender differences in dopaminergic function in striatum and nucleus accumbens. Pharmacol Biochem Behav. 1999;64(4):803‐812. 10593204 10.1016/s0091-3057(99)00168-9

[58] Bédard PJ , Malouin F , Dipaolo T , Labrie F . Estradiol, TRH and striatal dopaminergic mechanisms. Prog Neuropsychopharmacol Biol Psychiatry. 1982;6(4–6):555‐561. 6819600 10.1016/s0278-5846(82)80149-8

[59] Bosse R , DiPaolo T . The modulation of brain dopamine and GABAA receptors by estradiol: a clue for CNS changes occurring at menopause. Cell Mol Neurobiol. 1996;16(2):199‐212. 8743969 10.1007/BF02088176 PMC11563143

[60] Xiao L , Becker JB . Quantitative microdialysis determination of extracellular striatal dopamine concentration in male and female rats: effects of estrous cycle and gonadectomy. Neurosci Lett. 1994;180(2):155‐158. 7700570 10.1016/0304-3940(94)90510-x

[61] Russo G , Nieus TR , Maggi S , Taverna S . Dynamics of action potential firing in electrically connected striatal fast‐spiking interneurons. Front Cell Neurosci. 2013;7:209. 24294191 10.3389/fncel.2013.00209 PMC3827583

[62] Scofield MD , Heinsbroek JA , Gipson CD , et al. The nucleus Accumbens: mechanisms of addiction across drug classes reflect the importance of glutamate homeostasis. Pharmacol Rev. 2016;68(3):816‐871. 27363441 10.1124/pr.116.012484 PMC4931870

[63] Mulder AB , Hodenpijl MG , Lopes da Silva FH . Electrophysiology of the hippocampal and amygdaloid projections to the nucleus accumbens of the rat: convergence, segregation, and interaction of inputs. J Neurosci. 1998;18(13):5095‐5102. 9634575 10.1523/JNEUROSCI.18-13-05095.1998 PMC6792568

[64] O'Donnell P , Grace AA . Synaptic interactions among excitatory afferents to nucleus accumbens neurons: hippocampal gating of prefrontal cortical input. J Neurosci. 1995;15(5 Pt 1):3622‐3639. 7751934 10.1523/JNEUROSCI.15-05-03622.1995 PMC6578219

[65] Britt JP , Benaliouad F , McDevitt RA , Stuber GD , Wise RA , Bonci A . Synaptic and behavioral profile of multiple glutamatergic inputs to the nucleus accumbens. Neuron. 2012;76(4):790‐803. 23177963 10.1016/j.neuron.2012.09.040 PMC3607383

[66] Papp E , Borhegyi Z , Tomioka R , Rockland KS , Mody I , Freund TF . Glutamatergic input from specific sources influences the nucleus accumbens‐ventral pallidum information flow. Brain Struct Funct. 2012;217(1):37‐48. 21643647 10.1007/s00429-011-0331-z

[67] Sesack SR , Carr DB , Omelchenko N , Pinto A . Anatomical substrates for glutamate‐dopamine interactions: evidence for specificity of connections and extrasynaptic actions. Ann N Y Acad Sci. 2003;1003:36‐52. 14684434 10.1196/annals.1300.066

[68] Stuber GD , Sparta DR , Stamatakis AM , et al. Excitatory transmission from the amygdala to nucleus accumbens facilitates reward seeking. Nature. 2011;475(7356):377‐380. 21716290 10.1038/nature10194 PMC3775282

[69] Dorris DM , Cao J , Willett JA , Hauser CA , Meitzen J . Intrinsic excitability varies by sex in pre‐pubertal striatal medium spiny neurons. J Neurophysiol. 2015;113(3):720‐729. 25376786 10.1152/jn.00687.2014 PMC4312860

[70] Cao J , Dorris DM , Meitzen J . Neonatal masculinization blocks increased excitatory synaptic input in female rat nucleus Accumbens Core. Endocrinology. 2016;157(8):3181‐3196. 27285859 10.1210/en.2016-1160 PMC4967116

[71] Cao J , Willett JA , Dorris DM , Meitzen J . Sex differences in medium spiny neuron excitability and glutamatergic synaptic input: heterogeneity across striatal regions and evidence for estradiol‐dependent sexual differentiation. Front Endocrinol (Lausanne). 2018;9:173. 29720962 10.3389/fendo.2018.00173 PMC5915472

[72] Willett JA , Will T , Hauser CA , Dorris DM , Cao J , Meitzen J . No evidence for sex differences in the electrophysiological properties and excitatory synaptic input onto nucleus Accumbens Shell medium spiny neurons. eNeuro. 2016;3(1):pii: ENEURO.0147–15.2016. 10.1523/ENEURO.0147-15.2016 PMC4757778 27022621

[73] Willett JA , Cao J , Johnson A , Patel OH , Dorris DM , Meitzen J . The estrous cycle modulates rat caudate‐putamen medium spiny neuron physiology. Eur J Neurosci. 2020;52(1):2737‐2755. 31278786 10.1111/ejn.14506 PMC6943200

[74] Proaño SB , Krentzel AA , Meitzen J . Differential and synergistic roles of 17β‐estradiol and progesterone in modulating adult female rat nucleus accumbens core medium spiny neuron electrophysiology. J Neurophysiol. 2020;123(6):2390‐2405. 32401164 10.1152/jn.00157.2020 PMC7311720

[75] Proano SB , Morris HJ , Kunz LM , Dorris DM , Meitzen J . Estrous cycle‐induced sex differences in medium spiny neuron excitatory synaptic transmission and intrinsic excitability in adult rat nucleus accumbens core. J Neurophysiol. 2018;120(3):1356‐1373. 29947588 10.1152/jn.00263.2018 PMC6171053

[76] Forlano PM , Woolley CS . Quantitative analysis of pre‐ and postsynaptic sex differences in the nucleus accumbens. J Comp Neurol. 2010;518(8):1330‐1348. 20151363 10.1002/cne.22279 PMC2867251

[77] Wissman AM , McCollum AF , Huang GZ , Nikrodhanond AA , Woolley CS . Sex differences and effects of cocaine on excitatory synapses in the nucleus accumbens. Neuropharmacology. 2011;61:217‐227. 21510962 10.1016/j.neuropharm.2011.04.002 PMC3105198

[78] Wissman AM , May RM , Woolley CS . Ultrastructural analysis of sex differences in nucleus accumbens synaptic connectivity. Brain Struct Funct. 2012;217(2):181‐190. 21987050 10.1007/s00429-011-0353-6 PMC3275686

[79] Sazdanovic M , Slobodanka M , Zivanovic‐Macuzic I , et al. Sexual dimorphism of medium‐sized neurons with spines in human nucleus accumbens. Arch Biol Sci. 2013;65(3):1149‐1155.

[80] Peterson BM , Mermelstein PG , Meisel RL . Estradiol mediates dendritic spine plasticity in the nucleus accumbens core through activation of mGluR5. Brain Struct Funct. 2015;220(4):2415‐2422. 24878822 10.1007/s00429-014-0794-9 PMC5221506

[81] Staffend NA , Loftus CM , Meisel RL . Estradiol reduces dendritic spine density in the ventral striatum of female Syrian hamsters. Brain Struct Funct. 2011;215(3–4):187‐194. 20953625 10.1007/s00429-010-0284-7 PMC3057377

[82] Sherman BM , Korenman SG . Hormonal characteristics of the human menstrual cycle throughout reproductive life. J Clin Invest. 1975;55(4):699‐706. 1120778 10.1172/JCI107979 PMC301805

[83] Erskine MS . Solicitation behavior in the estrous female rat: a review. Horm Behav. 1989;23(4):473‐502. 2691387 10.1016/0018-506x(89)90037-8

[84] Hubscher CH , Brooks DL , Johnson JR . A quantitative method for assessing stages of the rat estrous cycle. Biotech Histochem. 2005;80(2):79‐87. 16195173 10.1080/10520290500138422

[85] Westwood FR . The female rat reproductive cycle: a practical histological guide to staging. Toxicol Pathol. 2008;36(3):375‐384. 18441260 10.1177/0192623308315665

[86] Beach FA . Sexual attractivity, proceptivity, and receptivity in female mammals. Horm Behav. 1976;7(1):105‐138. 819345 10.1016/0018-506x(76)90008-8

[87] Blaustein JD . Neuroendocrine regulation of feminine sexual behavior: lessons from rodent models and thoughts about humans. Annu Rev Psychol. 2008;59:93‐118. 17678443 10.1146/annurev.psych.59.103006.093556

[88] Kow LM , Pfaff DW . Effects of estrogen treatment on the size of receptive field and response threshold of pudendal nerve in the female rat. Neuroendocrinology. 1973;13(4):299‐313. 4779328 10.1159/000122214

[89] Micevych PE , Meisel RL . Integrating neural circuits controlling female sexual behavior. Front Syst Neurosci. 2017;11:42. 28642689 10.3389/fnsys.2017.00042 PMC5462959

[90] Adams C , Chen X , Moenter SM . Changes in GABAergic transmission to and intrinsic excitability of gonadotropin‐releasing hormone (GnRH) neurons during the Estrous Cycle in Mice. eNeuro. 2018;5(5):ENEURO.0171‐18.2018. 10.1523/ENEURO.0171-18.2018 PMC6223108 30417076

[91] Shansky RM . Are hormones a “female problem” for animal research? Science. 2019;364(6443):825‐826. 31147505 10.1126/science.aaw7570

[92] Prendergast BJ , Onishi KG , Zucker I . Female mice liberated for inclusion in neuroscience and biomedical research. Neurosci Biobehav Rev. 2014;40C:1‐5. 10.1016/j.neubiorev.2014.01.001 24456941

[93] Yoest KE , Cummings JA , Becker JB . Estradiol, dopamine and motivation. Cent Nerv Syst Agents Med Chem. 2014;14(2):83‐89. 25540977 10.2174/1871524914666141226103135 PMC4793919

[94] Blume SR , Freedberg M , Vantrease JE , et al. Sex‐ and estrus‐dependent differences in rat basolateral amygdala. J Neurosci. 2017;37(44):10567‐10,586. 28954870 10.1523/JNEUROSCI.0758-17.2017 PMC5666581

[95] Olmos G , Naftolin F , Perez J , Tranque PA , Garcia‐Segura LM . Synaptic remodeling in the rat arcuate nucleus during the estrous cycle. Neuroscience. 1989;32(3):663‐667. 2601838 10.1016/0306-4522(89)90288-1

[96] Terasawa E , Timiras PS . Electrical activity during the estrous cycle of the rat: cyclic changes in limbic structures. Endocrinology. 1968;83(2):207‐216. 4874282 10.1210/endo-83-2-207

[97] Woolley CS , McEwen BS . Roles of estradiol and progesterone in regulation of hippocampal dendritic spine density during the estrous cycle in the rat. J Comp Neurol. 1993;336(2):293‐306. 8245220 10.1002/cne.903360210

[98] Schulz DJ . Plasticity and stability in neuronal output via changes in intrinsic excitability: it's what's inside that counts. J Exp Biol. 2006;209(Pt 24):4821‐4827. 17142671 10.1242/jeb.02567

[99] Dong Y , Green T , Saal D , et al. CREB modulates excitability of nucleus accumbens neurons. Nat Neurosci. 2006;9(4):475‐477. 16520736 10.1038/nn1661

[100] Ferguson SM , Eskenazi D , Ishikawa M , et al. Transient neuronal inhibition reveals opposing roles of indirect and direct pathways in sensitization. Nat Neurosci. 2011;14(1):22‐24. 21131952 10.1038/nn.2703 PMC3058296

[101] Grillner S , Hellgren J , Menard A , Saitoh K , Wikstrom MA . Mechanisms for selection of basic motor programs‐‐roles for the striatum and pallidum. Trends Neurosci. 2005;28(7):364‐370. 15935487 10.1016/j.tins.2005.05.004

[102] Kourrich S , Thomas MJ . Similar neurons, opposite adaptations: psychostimulant experience differentially alters firing properties in accumbens core versus shell. J Neurosci. 2009;29(39):12275‐12283. 19793986 10.1523/JNEUROSCI.3028-09.2009 PMC3307102

[103] Mu P , Moyer JT , Ishikawa M , et al. Exposure to cocaine dynamically regulates the intrinsic membrane excitability of nucleus accumbens neurons. J Neurosci. 2010;30(10):3689‐3699. 20220002 10.1523/JNEUROSCI.4063-09.2010 PMC2853189

[104] Tan CL , Plotkin JL , Veno MT , et al. MicroRNA‐128 governs neuronal excitability and motor behavior in mice. Science. 2013;342(6163):1254‐1258. 24311694 10.1126/science.1244193 PMC3932786

[105] Werme M , Messer C , Olson L , et al. Delta FosB regulates wheel running. J Neurosci. 2002;22(18):8133‐8138. 12223567 10.1523/JNEUROSCI.22-18-08133.2002 PMC6758121

[106] Proaño SB , Meitzen J . Estradiol decreases medium spiny neuron excitability in female rat nucleus accumbens core. J Neurophysiol. 2020;123(6):2465‐2475. 32432511 10.1152/jn.00210.2020 PMC7311729

[107] Scharfman HE , Hintz TM , Gomez J , et al. Changes in hippocampal function of ovariectomized rats after sequential low doses of estradiol to simulate the preovulatory estrogen surge. Eur J Neurosci. 2007;26(9):2595‐2612. 17970745 10.1111/j.1460-9568.2007.05848.x PMC2225429

[108] Gross KS , Mermelstein PG . Estrogen receptor signaling through metabotropic glutamate receptors. Vitam Horm. 2020;114:211‐232. 32723544 10.1016/bs.vh.2020.06.003

[109] Zakon HH . The effects of steroid hormones on electrical activity of excitable cells. Trends Neurosci. 1998;21(5):202‐207. 9610884 10.1016/s0166-2236(97)01209-5

[110] Constantin S , Moenter SM , Piet R . The electrophysiologic properties of gonadotropin‐releasing hormone neurons. J Neuroendocrinol. 2021;e13073. Online ahead of print. 34939256 10.1111/jne.13073 PMC9163209

[111] Vail G , Roepke TA . Membrane‐initiated estrogen signaling via Gq‐coupled GPCR in the central nervous system. Steroids. 2019;142:77‐83. 29378226 10.1016/j.steroids.2018.01.010 PMC6064680

[112] Krentzel AA , Barrett LR , Meitzen J . Estradiol rapidly modulates excitatory synapse properties in a sex‐ and region‐specific manner in rat nucleus accumbens core and caudate‐putamen. J Neurophysiol. 2019;122(3):1213‐1225. 31314648 10.1152/jn.00264.2019 PMC6766735

[113] Peterson BM , Martinez LA , Meisel RL , Mermelstein PG . Estradiol impacts the endocannabinoid system in female rats to influence behavioral and structural responses to cocaine. Neuropharmacology. 2016;110:118‐124. 27266915 10.1016/j.neuropharm.2016.06.002 PMC5028287

[114] Meitzen J , Pflepsen KR , Stern CM , Meisel RL , Mermelstein PG . Measurements of neuron soma size and density in rat dorsal striatum, nucleus accumbens core and nucleus accumbens shell: differences between striatal region and brain hemisphere, but not sex. Neurosci Lett. 2011;487(2):177‐181. 20951763 10.1016/j.neulet.2010.10.017 PMC3004999

[115] Wong JE , Cao J , Dorris DM , Meitzen J . Genetic sex and the volumes of the caudate‐putamen, nucleus accumbens core and shell: original data and a review. Brain Struct Funct. 2016;221(8):4257‐4267. 26666530 10.1007/s00429-015-1158-9

[116] Becker JB , Rudick CN . Rapid effects of estrogen or progesterone on the amphetamine‐induced increase in striatal dopamine are enhanced by estrogen priming: a microdialysis study. Pharmacol Biochem Behav. 1999;64(1):53‐57. 10494997 10.1016/s0091-3057(99)00091-x

[117] Krentzel AA , Proano S , Patisaul HB , Meitzen J . Temporal and bidirectional influences of estradiol on voluntary wheel running in adult female and male rats. Horm Behav. 2020;120:104694. 31978389 10.1016/j.yhbeh.2020.104694 PMC7117976

[118] Miller CK , Krentzel AA , Patisaul HB , Meitzen J . Metabotropic glutamate receptor subtype 5 (mGlu5) is necessary for estradiol mitigation of light‐induced anxiety behavior in female rats. Physiol Behav. 2020;214:112770. 31830486 10.1016/j.physbeh.2019.112770 PMC7029786

[119] Turrigiano G . Homeostatic synaptic plasticity: local and global mechanisms for stabilizing neuronal function. Cold Spring Harb Perspect Biol. 2012;4(1):a005736. 22086977 10.1101/cshperspect.a005736 PMC3249629

[120] Tien NW , Kerschensteiner D . Homeostatic plasticity in neural development. Neural Dev. 2018;13(1):9. 29855353 10.1186/s13064-018-0105-x PMC5984303

[121] Tonn Eisinger KR , Larson EB , Boulware MI , Thomas MJ , Mermelstein PG . Membrane estrogen receptor signaling impacts the reward circuitry of the female brain to influence motivated behaviors. Steroids. 2018;133:53‐59. 29195840 10.1016/j.steroids.2017.11.013 PMC5864533

[122] Jenkins WJ , Becker JB . Role of the striatum and nucleus accumbens in paced copulatory behavior in the female rat. Behav Brain Res. 2001;121(1–2):119‐128. 11275289 10.1016/s0166-4328(00)00394-6

[123] Meisel RL , Mullins AJ . Sexual experience in female rodents: cellular mechanisms and functional consequences. Brain Res. 2006;1126(1):56‐65. 16978593 10.1016/j.brainres.2006.08.050 PMC1779900

[124] Miller CK , Halbing AA , Patisaul HB , Meitzen J . Interactions of the estrous cycle, novelty, and light on female and male rat open field locomotor and anxiety‐related behaviors. Physiol Behav. 2021;228:113203. 33045240 10.1016/j.physbeh.2020.113203 PMC7736204

[125] Krentzel AA , Kimble LC , Dorris DM , Horman BM , Meitzen J , Patisaul HB . FireMaster® 550 (FM 550) exposure during the perinatal period impacts partner preference behavior and nucleus accumbens core medium spiny neuron electrophysiology in adult male and female prairie voles, Microtus ochrogaster. Horm Behav. 2021;134:105019. 34182292 10.1016/j.yhbeh.2021.105019 PMC8403633

[126] Willett JA , Johnson AG , Vogel AR , Patisaul HB , McGraw LA , Meitzen J . Nucleus accumbens core medium spiny neuron electrophysiological properties and partner preference behavior in the adult male prairie vole, Microtus ochrogaster. J Neurophysiol. 2018;119(4):1576‐1588. 29361665 10.1152/jn.00737.2017 PMC5966737

[127] Cao J , Dorris DM , Meitzen J . Electrophysiological properties of medium spiny neurons in the nucleus accumbens core of prepubertal male and female Drd1a‐tdTomato line 6 BAC transgenic mice. J Neurophysiol. 2018;120(4):1712‐1727. 29975170 10.1152/jn.00257.2018 PMC6230806

[128] Willett JA , Cao J , Dorris DM , Johnson AG , Ginnari LA , Meitzen J . Electrophysiological properties of medium spiny neuron subtypes in the caudate‐putamen of Prepubertal male and female Drd1a‐tdTomato line 6 BAC transgenic mice. eNeuro. 2019;6(2):pii: ENEURO.0016–19.2019. 10.1523/ENEURO.0016-19.2019 PMC6426437 30899778

[129] Kupchik YM , Brown RM , Heinsbroek JA , Lobo MK , Schwartz DJ , Kalivas PW . Coding the direct/indirect pathways by D1 and D2 receptors is not valid for accumbens projections. Nat Neurosci. 2015;18(9):1230‐1232. 26214370 10.1038/nn.4068 PMC4551610

[130] Delevich K , Hall CD , Wilbrecht L . Prepubertal ovariectomy alters dorsomedial striatum indirect pathway neuron excitability and explore/exploit balance in female mice. bioRxiv. 2021; Preprint.10.1016/j.yhbeh.2026.10599542790140

[131] Quigley JA , Becker JB . Activation of G‐protein coupled estradiol receptor 1 in the dorsolateral striatum attenuates preference for cocaine and saccharin in male but not female rats. Horm Behav. 2021;130:104949. 33609527 10.1016/j.yhbeh.2021.104949 PMC8012250

[132] Oberlander JG , Woolley CS . 17β‐estradiol acutely potentiates glutamatergic synaptic transmission in the hippocampus through distinct mechanisms in males and females. J Neurosci. 2016;36(9):2677‐2690. 26937008 10.1523/JNEUROSCI.4437-15.2016 PMC4879212

[133] Becker JB , Snyder PJ , Miller MM , Westgate SA , Jenuwine MJ . The influence of estrous cycle and intrastriatal estradiol on sensorimotor performance in the female rat. Pharmacol Biochem Behav. 1987;27(1):53‐59. 3615546 10.1016/0091-3057(87)90476-x

[134] Tansey EM , Arbuthnott GW , Fink G , Whale D . Oestradiol‐17 beta increases the firing rate of antidromically identified neurones of the rat neostriatum. Neuroendocrinology. 1983;37(2):106‐110. 6684218 10.1159/000123527

[135] Arnauld E , Dufy B , Pestre M , Vincent JD . Effects of estrogens on the responses of caudate neurons to microiontophoretically applied dopamine. Neurosci Lett. 1981;21(3):325‐331. 7219879 10.1016/0304-3940(81)90225-1

[136] Cao J , Meitzen J . Perinatal activation of ERα and ERβ but not GPER‐1 masculinizes female rat caudate‐putamen medium spiny neuron electrophysiological properties. J Neurophysiol. 2021;125(6):2322‐2338. 33978486 10.1152/jn.00063.2021 PMC8285660

[137] Lipscombe D , Helton TD , Xu W . L‐type calcium channels: the low down. J Neurophysiol. 2004;92(5):2633‐2641. 15486420 10.1152/jn.00486.2004

[138] Hu M , Watson CJ , Kennedy RT , Becker JB . Estradiol attenuates the K+‐induced increase in extracellular GABA in rat striatum. Synapse. 2006;59(2):122‐124. 16320305 10.1002/syn.20221

[139] Tozzi A , de Iure A , Tantucci M , et al. Endogenous 17beta‐estradiol is required for activity‐dependent long‐term potentiation in the striatum: interaction with the dopaminergic system. Front Cell Neurosci. 2015;9:192. 26074768 10.3389/fncel.2015.00192 PMC4445326

[140] Remage‐Healey L . Brain estrogen signaling effects acute modulation of acoustic communication behaviors: a working hypothesis. Bioessays. 2012;34:1009‐1016. 23065844 10.1002/bies.201200081 PMC3710781

[141] Brenowitz EA , Lent K . Afferent input is necessary for seasonal growth and maintenance of adult avian song control circuits. J Neurosci. 2001;21(7):2320‐2329. 11264307 10.1523/JNEUROSCI.21-07-02320.2001 PMC6762386

[142] Brenowitz EA , Lent K . Act locally and think globally: intracerebral testosterone implants induce seasonal‐like growth of adult avian song control circuits. Proc Natl Acad Sci U S A. 2002;99(19):12421‐12426. 12218180 10.1073/pnas.192308799 PMC129460

[143] Euvrard C , Labrie F , Boissier JR . Effect of estrogen on changes in the activity of striatal cholinergic neurons induced by DA drugs. Brain Res. 1979;169(1):215‐220. 455094 10.1016/0006-8993(79)90392-5

[144] Labonté B , Engmann O , Purushothaman I , et al. Sex‐specific transcriptional signatures in human depression. Nat Med. 2017;23(9):1102‐1111. 28825715 10.1038/nm.4386 PMC5734943

[145] Johnson AR , Thibeault KC , Lopez AJ , et al. Cues play a critical role in estrous cycle‐dependent enhancement of cocaine reinforcement. Neuropsychopharmacology. 2019;44(7):1189‐1197. 30728447 10.1038/s41386-019-0320-0 PMC6785030

